# Cannabis use and depression: a longitudinal study of a national cohort of Swedish conscripts

**DOI:** 10.1186/1471-244X-12-112

**Published:** 2012-08-16

**Authors:** Edison Manrique-Garcia, Stanley Zammit, Christina Dalman, Tomas Hemmingsson, Peter Allebeck

**Affiliations:** 1 Department of Public Health Sciences, Division of Social Medicine, Karolinska Institutet, Norrbacka floor 6, Stockholm,, S-17176, Sweden; 2 Department of Psychological Medicine & Neurology, MRC Centre for Neuropsychiatric Genetics and Genomics, Cardiff University, Henry Wellcome Building, Cardiff, CF14 4XN, UK; 3 Department of Public Health Sciences, Division of Public Health Epidemiology, Karolinska Institutet, Norrbacka floor 7, Stockholm,, S-17176, Sweden; 4 Department of Public Health Sciences, Division of Occupational and Environmental Medicine, Karolinska Institutet, Norrbacka floor 4, Stockholm,, S-17176, Sweden

**Keywords:** Cannabis, Depression, Schizoaffective disorder

## Abstract

**Background:**

While there is increasing evidence on the association between cannabis use and psychotic outcomes, it is still unclear whether this also applies to depression. We aim to assess whether risk of depression and other affective outcomes is increased among cannabis users.

**Methods:**

A cohort study of 45 087 Swedish men with data on cannabis use at ages 18–20. Diagnoses of unipolar disorder, bipolar disorder, affective psychosis and schizoaffective disorder were identified from inpatient care records over a 35-year follow-up period. Cox proportional hazard modeling was used to assess the hazard ratio (HR) of developing these disorders in relation to cannabis exposure.

**Results:**

Only subjects with the highest level of cannabis use had an increased crude hazard ratio for depression (HR 1.5, 95% confidence interval (CI), 1.0-2.2), but the association disappeared after adjustment for confounders. There was a strong graded association between cannabis use and schizoaffective disorder, even after control for confounders, although the numbers were small (HR 7.4, 95% CI, 1.0-54.3).

**Conclusion:**

We did not find evidence for an increased risk of depression among those who used cannabis. Our finding of an increased risk of schizoaffective disorder is consistent with previous findings on the relation between cannabis use and psychosis.

## Background

While there is increasing evidence on the association between cannabis use and psychotic outcomes [1], it is still unclear whether this also applies to other psychiatric disorders, especially depression. Several clinical studies and case reports have found an association between cannabis use and subsequent depression, but the causal direction of the association has been difficult to establish [2-4].

A review by Degenhardt et al. [5] found a modest association between heavy or problematic cannabis use and depression, but little evidence for an association between depression and infrequent cannabis use [5]. A review by Moore et al. [6] identified ten cohort studies that examined diagnoses of depression, of which five reported evidence of an association with cannabis use that persisted after adjustment for confounding. However, two of these five studies found significant associations only in sub-group analyses, while the range of potentially important confounders adjusted for in the majority of the studies was rather limited. Moreover, only four of the ten cohort studies excluded participants with affective symptoms at baseline. Similarly inconsistent findings were reported in a multinational study by de Graaf et al. [7], which reported a modest overall association between cannabis use and later risk of depression, but also showed that the nature of the association is not consistent between countries.

Degenhardt et al. [5] and Moore et al. [6] concluded that findings on the association between cannabis and depression were inconsistent, probably due to small samples and problems related to reverse causality. The inconsistency of findings across the different studies may also be due to the use of different measures of cannabis use as well as affective outcomes [8] and inadequate consideration of confounding [6]. Risk factors for depression include exposure to adverse life events, presence of other mental health problems, socio-economic adversity, substance use and problematic peer relationships [9,10]. Since these factors are also associated with cannabis use [5,6,11], control for these is important.

Psychotic symptoms are present not only in schizophrenia, but also in affective psychoses and bipolar disorders. The evidence that cannabis use increases the risk of psychotic symptoms implies that there may also be associations between cannabis and affective disorders with psychotic features, such as schizoaffective and bipolar disorders. Moreover, epidemiological and genetic molecular studies have found evidence that schizophrenia and bipolar disorder share some common genetic causes [12,13]. There may also be other risk factors in common across these disorders, such as cannabis use. Therefore, while the diagnosis of schizoaffective disorder is controversial [14], we find it of interest to assess whether cannabis consumption might increase its occurrence. Another specific outcome of interest is manic or hypomanic episodes, for which an association with cannabis use has been found [15].

Different mechanisms have been proposed to explain a possible association between cannabis use and depression. In animal experiments, long-term exposure to cannabinoids has been found to alter the activity of 5-HT serotonin receptors [16]. There is strong evidence that disruptions to the serotonin system at least partly underlie the pathophysiological mechanisms that lead to depression [16]. It has been suggested that large doses of delta-9-tetrahydrocannabinol, the main psychoactive component of cannabis, can, in the longer-term, result in depression through the effects of THC on serotonin and other neurotransmitters [5]. However, as yet, there is no animal model to support this hypothesis. Long-term heavy cannabis use has also been associated with significant bilateral reduction in hippocampal and amygdala volumes [17]. Given the association between depression and hippocampal volume reduction [18], it is feasible that depressive symptoms occur as a consequence of structural brain changes following long-term cannabis use.

It has also been hypothesized that heavy cannabis use may precipitate depression indirectly by reducing educational attainment, earning capacity and quality of social relationships through its effects on cognition and motivation [5].

The Swedish conscript survey is, to date, the largest population-based cohort study with data on cannabis use, and also on a number of social and personality background factors. The conscript cohort has been used previously to examine the association between cannabis and schizophrenia [19,20], and cannabis and suicide [21]. The aim of the present study was to address the association between cannabis and depression. Although depression is a common condition, diagnostic data was available from inpatient care only, and it was therefore not possible to examine associations across the whole spectrum of depressive disorders. Accordingly, we focus here on depressive states severe enough to warrant hospital admission. Our specific aims were to examine:

whether there is an increased risk of depression among cannabis users compared with non-users;

whether any such association differs according to the affective outcome in question;

whether there is an association between cannabis and schizoaffective disorder

## Methods

### Study population

The data used in this study were derived from a nationwide survey of 50 087 Swedish men, who were examined for compulsory military training in 1969–1970. Conscription examination was compulsory for all young Swedish men until recently. However, this cohort is the only one that has retained personal identification on matters related to drug use and other behavioral characteristics, thereby enabling record linkage with Sweden’s National Inpatient Register. Over 98% of the men were aged 18–20 years at conscription, and only 2–3% of them were exempted from conscription, mainly due to a severe mental or physical handicap or a congenital disorder. The conscription procedure took 1½ days for each subject to complete. All were given an IQ test and responded to two questionnaires. The first questionnaire concerned social background, upbringing conditions, friendship, relationships, attitudes, and adjustment at school and work. The second concerned use of alcohol, tobacco and other substances. In addition, all the conscripts underwent a medical examination, and also a structured interview with a psychologist. The ones who reported or presented psychiatric problems were referred to a psychiatrist, and any psychiatric disorders found was diagnosed according to the International Classification of Diseases (8^th^ revision).

A total of 607 subjects with depression (ICD-8; 3004) and 30 with psychosis (ICD-8; 295, 296, 297, 298) were identified during conscription, and they were therefore excluded from the analysis. In order to avoid misclassification of outcome, 11 subjects who had diagnoses of both depression and either schizoaffective disorder or schizophrenia during follow-up were also excluded. Due to missing information on cannabis use, we excluded a further 3 614 individuals. The final analytical sample consisted of 45 087 subjects, born 1949–51.

Permission to use the conscription database for research purposes and to perform the relevant record linkages was granted by the Stockholm Regional Ethical Review Board.

### Exposure

Information on use of cannabis and other drugs was obtained from the conscription questionnaire on alcohol, tobacco and substance use. The questions covered whether subjects had ever used drugs, which drugs had been used from a list of alternatives, the first drug used, the drug most commonly used, and how many times a drug had been used.

The main cannabis measure that we employed was reported level of cannabis use as categorized in the response options in the questionnaire: Never, Once, 2–4, 5–10, 11–50, >50. However, due to the small numbers of cases in the sub-groups analyses, we compared outcomes for those having ever used cannabis (thus collapsing all subjects who reported cannabis use into one category) with those who had never used cannabis, and also compared outcomes for those reporting highest level of use (‘>50 times’) with those who had never used cannabis.

### Outcomes and follow-up

The Swedish National Inpatient Register, which records all inpatient admissions to hospitals in Sweden, was used to identify admissions for selected diagnoses from 1973 until 2007. The Swedish register recorded approximately 83% of all psychiatric admissions in 1973, 97% in 1974–1983, and 95% in 1984–1986, and has been virtually complete since 1987. Diagnoses were coded according to the Swedish version of the ICD (ICD-8 during 1965–1986, ICD-9 1987–1996, ICD-10 1997–2007), and divided into the following diagnostic groups: 1. Unipolar depression, 2. Bipolar disorder and affective psychosis, 3. Schizoaffective disorder.

Unipolar depression:

ICD 8: Depressive neurosis (300.40)

ICD 9: Depressive disorder not elsewhere classified (311), neurotic depression (300E)

ICD 10: Depressive episode (F32) excluding 323, and recurrent depressive disorder (F33) excluding 333

Bipolar disorder and affective psychosis:

ICD 8:

Affective psychosis consisting of involutional melancholia (296.00)

Manic-depression psychosis consisting of manic type (296.10), depressed type (296,20), circular type (296.30), other (296.88) and unspecified (296.99)

ICD 9:

Unipolar affective psychosis consisting of manic or hypomanic episodes (296A), melancholia (296B)

Bipolar affective psychosis consisting of manic type (296 C), melancholia type (296D), mixed form (296E), other (296 W) and unspecified (296X)

ICD 10:

Manic episode (F30), bipolar affective disorder (F31), severe depressive episode with psychotic symptoms (F323), and recurrent depressive disorder with current psychotic symptoms (F333)

*Schizoaffective disorder*:

ICD 8: Schizophrenia, type schizoaffective (295.0)

ICD 9: Schizoaffective form (295 H)

ICD 10: Schizoaffective disorders (F25).

Furthermore, we conducted a specific analysis for the diagnoses of mania, which included the following diagnostic codes:

ICD 8: Manic-depressive psychosis (296.10).

ICD 9: Bipolar affective psychosis, manic type (296 C).

ICD 10: Manic episode (F30, F31.0, F31.1, F31.2)

Data were linked to Sweden’s National Cause of Death Register and the Swedish Migration Register. About 1 300 individuals emigrated from Sweden, and 2 620 died during the follow-up period. The date of first emigration and day of death were used as censoring points. First diagnoses of any of the outcomes above were used as the primary end-points. The mean follow-up period from conscription to censoring of data was 32 years, with a range of 1 day to 35 years.

### Possible confounders

We selected potential confounding variables on the basis of prior research indicating that they are likely to be associated with both cannabis use and affective outcomes. Relevant variables were obtained from the conscription questionnaires and psychological assessments.

a) Diagnosis of personality disorders assessed by a psychiatrist at conscription: any vs. none.

b) IQ score consisted of four main subtests parts (verbal IQ, visuospatial ability, general knowledge and mechanical ability); these four subtests were aggregated to give an overall standardized intelligence score, ranging 1 to 9 (< 74, 74 to 81, 82 to 89, 90 to 95, 96 to 104, 105 to 110, 111 to 118, 119 to 126, > 126). The IQ test has been described elsewhere in detail [22,23].We further transformed the standard-nine values into a composite standard-three scale: highest (111 to > 126) vs. middle (90 to 110), lowest (< 74 to 89).

c) The variable “Disturbed behavior in childhood” was derived from questions on truancy from school (once a week, once a month, once per term, occasionally), having been in contacts with police and childcare authorities (once, more, never), running away from home (once, more, never), and having been sanctioned in school (once, more, never). These four questions were aggregated to give an overall standardized composite score ranging from 0–9: very low (0 to 1) vs. low (2 to 3), average (4 to 5), high (6 to 7), very high (8 to 9).

d) The variable “Social adjustment” was derived from questions on popularity at school (1 = very popular to 5 = unpopular), number of close friends (> 5 to none), being in a relationship with a girl (more than a year, several months, one month or less, no). These three questions were aggregated to give an overall standardized score ranges from 0–11: very good (0 to 2) vs. good (3 to 5), low (6 to 8), very low (9 to 11).

e) The variable “Risky use of alcohol” was derived from questions on high consumption of alcohol: none vs. at least one of the following indicators– consumption of at least 250 g 100% alcohol/week; have taken an eye-opener during a hangover; have been apprehended for drunkenness; have reported being drunk often.

f) The variable “Smoking” was based on self-reported information from questionnaires: 1= >20 cigarettes/day; 2 = 11–20 cigarettes/day; 3 = 1–10 cigarettes/day; 4 = non-smokers.

g) “Early adulthood socioeconomic position (Early adulthood SEP)” was based on information from Statistics Sweden on occupation: 1) high/intermediate nonmanual 2) low nonmanual 3) manual skill/unskilled 4) farmers/self-employed/unclassified.

h) “Use of other drugs” (Mebumal, Opium, Preludin, Morphine) was based on information from questionnaires: any vs. none.

i) “Brought up in a city” was based on self-reported information on upbringing: Rural vs. city with less than 50 000 inhabitants, city with more than 50 000 inhabitants, any one of Sweden’s three large metropolitan areas (Stockholm, Gothenburg, and Malmö).

### Statistical analyses

First, the overall depression was defined as first hospital admission for any diagnosis of unipolar depression, bipolar disorder or affective psychosis. Second, specific analyses were performed for unipolar depression, and bipolar disorder and affective psychosis. Due to variation in the ICD coding system over the years, we placed both bipolar disorder and affective psychosis in the same category. Third, an analysis was performed on the association between cannabis use and schizoaffective disorder. Fourth, we performed a specific analysis of the association between cannabis use and manic disorder.

Cox proportional hazard modeling was used to assess the relative risks of developing the outcomes in relation to cannabis exposure. We explored the effect of each individual confounder on the relationship between cannabis use and each outcome. Crude and adjusted hazard ratio (HR), with 95% confidence interval (CI), was computed by level of cannabis use, and also for each potential confounder. We assessed the proportional hazard assumption between cannabis use and each outcome by using a Kaplan-Meier plot. We tested the equality across strata of each individual confounder to explore whether or not to include them in the final model. For the categorical variables we used the log-rank test of equality across strata; in fact, all the variables listed as possible confounders were retained. The quality of the model was tested by running a logistic regression and calculating Hosmer-Lemeshow’s GOF test which had a value was 0.55, showing that our observed results match the expected of the model population. The analyses were performed in SAS 9.1 for Windows.

## Results

Among the 45 087 subjects, a total of 1 146 individuals had depression (2.5%) during the 35 years of follow-up, and about 14% had used cannabis by age 18 to 20. Table 1 shows hazard ratios for depression (any case of unipolar disorder, bipolar disorder, or affective psychosis) by level of reported cannabis use. No association was found between frequency of cannabis use and risk of depression. Only subjects with the highest level of cannabis use had an increased crude hazard ratio for depression, but this association disappeared after adjustment for the confounders. Individual adjustment for each confounder showed that the variable that most contributed to reducing the hazard ratio was the composite variable ‘disturbed behavior in childhood’, which reduced the hazard ratio to one (data not shown).

**Table 1:** Hazard ratios for overall depression by reported frequency of cannabis use

**Cannabis use**	**No. exposed**	**No. cases**	**HR Crude**	HR adjusted*
Never	39 978	990	1	1
Once	1 202	28	0.9 (0.6-1.4)	0.9 (0.6-1.5)
2–4	1 486	51	1.4 (1.1-1.9)	1.2 (0.8-1.8)
5–10	839	24	1.2 (0.7-1.8)	1.1 (0.6-1.8)
11–50	727	24	1.3 (0.8-1.9)	0.6 (0.3-1.2)
>50	855	29	1.5 (1.0-2.2)	0.8 (0.4-1.5)
TOTAL	45 087	1 146		

* Adjustments for: prior personality disorders at conscription, IQ, disturbed behavior in childhood, social adjustment, risky use of alcohol, smoking, early adulthood socioeconomic position, use of other drugs, brought up in a city.

Table 2 shows the hazard ratios for the two outcomes unipolar disorder and bipolar disorder/affective psychosis, by category of cannabis use. The heaviest cannabis use (>50 times) was associated with an increased risk of unipolar depression (HR 1.8, 95% CI, 1.2-2.7), but this association was eliminated by adjustment for confounding. Disturbed behavior in childhood was again the confounder that most attenuated the hazard ratios (data not shown).

**Table 2:** Associations between cannabis use and affective outcomes

	**No. exposed**	**No. cases**	**HR Crude**	HR adjusted*
**BIPOLAR DISORDER/AFFECTIVE PSYCHOSIS**				
Never used cannabis	39 978	335	1	1
Ever used cannabis	5 109	45	1.1 (0.7-1.4)	1.1 (0.7-1.7)
>50 times	855	5	0.8 (0.3-1.8)	0.3 (0.1-2.2)
**UNIPOLAR DISORDER**				
Never used cannabis	39 978	655	1	1
Ever used cannabis	5 109	111	1.3 (1.1-1.6)	1.0 (0.7-1.2)
>50 times	855	24	1.8 (1.2-2.7)	0.9 (0.5-1.6)

* Adjustments for: prior personality disorders at conscription, IQ, disturbed behavior in childhood, social adjustment, risky use of alcohol, smoking, early adulthood socioeconomic position, use of other drugs, brought up in a city. The category “Ever used cannabis” includes all individuals who reported cannabis use, including those who reported “>50 times”.

Table 3 shows the hazard ratios for schizoaffective disorder by levels of cannabis use. There was a strong graded association between cannabis use and schizoaffective disorder. Even after control for confounding, there was a more than six-fold increased hazard ratio for subjects with the highest consumption level of cannabis (>50 times) in comparison with those who had never used cannabis.

**Table 3:** Association between cannabis use and schizoaffective disorder

**SCHIZOAFFECTIVE**	**No. exposed**	**No. cases**	**HR Crude**	HR adjusted*
Never used cannabis	39 978	47	1	1
Ever used cannabis	5 109	12	2.1 (1.1-3.8)	0.8 (0.2-2.9)
>50 times	855	7	7.5 (3.4-16.7)	7.4 (1.0-54.3)

* Adjustments for: prior personality disorders at conscription, IQ, disturbed behavior in childhood, social adjustment, risky use of alcohol, smoking, early adulthood socioeconomic position, use of other drugs, brought up in a city. The category “Ever used cannabis” includes all individuals who reported cannabis use, including those who reported “>50 times”.

A total of 118 cases of mania were identified in the full sample. Eight cases were found among subjects who had used cannabis (all levels of use), and one case among those with the highest consumption level. The hazard ratio for manic disorder of those who had used cannabis, compared with non-users, was 0.96 (95% CI, 0.4-1.9).

Table 4 shows the contribution of the different confounders to the outcome depression, crude and adjusted. Disturbed behavior was the variable most strongly associated with later depression, even after control for other variables (HR 2.7, 95% CI, 1.3-5.4). Also IQ, social adjustment and prior personality disorder were strong predictors.

**Table 4:** Risk of depression by potential confounders

**POTENTIAL CONFOUNDERS**	**HR Crude Analysis**	HR adjusted for other potential confounders^*^
Prior personality disorders (any vs. none)^a^	2.6 (2.0-3.4)	1.8 (1.2-2.5)
IQ score (lowest vs. highest)^1^	2.9 (2.4-3.4)	2.2 (1.7-2.8)
Risky use of alcohol indicators (none vs. at least one)^b^	1.7 (1.5-2.0)	1.1 (0.9-1.4)
Smoking (>20 cigarettes/day vs. non-smokers)^2^	2.6 (2.0-3.3)	1.7 (1.2-2.4)
Disturbed behavior in childhood (very high vs. very low)^3^	3.8 (2.1-6.8)	2.7 (1.3-5.4)
Social adjustment (very low vs. very good)^4^	2.2 (1.3-3.6)	2.0 (1.0-3.9)
Early adulthood SEP (manual skill/unskilled vs. high/intermediate nonmanual)^5^	1.6 (1.3-2.0)	1.2 (1.0-1.5)
Use of other drugs (any vs. none)^c^	0.8 (0.3-2.3)	0.6 (0.2-2.6)
Brought up in a city (metropolitan areas vs. rural)^6^	0.9 (0.8-1.1)	0.8 (0.6-1.1)

* adjusted for all potential confounders and cannabis use.

^a, b, c^ dichotomous variable.

^1, 2, 3, 4, 5, 6^ categorical variables, only one exposure group presented in the table.

## Discussion

Our main finding was that, after control for confounding factors and especially markers of disturbed behavior during childhood, there was no increased risk of future depression among cannabis users at age 18 to 20. With the large number of cases, and control for important background factors, we believe our study adds to previous findings supporting the hypothesis that cannabis use does not increase the risk of depression [24-26].

Cannabis use has also been associated with suicide [27]. However another study using the same longitudinal population as our study, found no association between cannabis and subsequent risk of completed suicide after adjusting for confounders [21]. Given the strong association between severe depression and suicide, our findings are consistent with those of Price et al. [21].

In contrast to a previous study [15], we did not find any increased risk of manic disorder associated with cannabis use. However the number of cases was small and particular caution is needed in drawing any conclusions from this result.

We found a strong association between history of cannabis use and schizoaffective disorder, which persisted after adjusting for confounders. Other studies have not been able to assess the effect of cannabis specifically on schizoaffective disorder, but have examined a broader spectrum of schizophrenia spectrum disorders [6]. The finding of a substantially increased risk of schizoaffective disorder among cannabis users is consistent with previous findings on the associations between cannabis and different schizophrenia-related disorders [6,28].

### Limitations

Identification of diagnoses of depression was limited to inpatient care, which means that our findings may not be applicable to milder forms of depression that do not require inpatient care. The diagnosis schizoaffective disorder has questionable validity as a separate clinical entity [14], but is often made when psychotic features are prominent, so we found it important to analyze separately. In this category, we only included those subjects with a diagnosis of schizoaffective disorder who did not also have a diagnosis of depression or schizophrenia during follow-up, to minimize overlapping diagnoses. Regarding the validity of diagnoses in Sweden’s National Inpatient Register, several studies have demonstrated adequate validity of major psychiatric diagnoses for epidemiological studies [29,30]. Information about cannabis use was restricted to data on the use of drugs by individuals up to time of conscription. However, consumption generally declines with age [31], so it is unlikely that the consumption pattern would change over time to the extent that potentially bias the association between cannabis use and depression. Although misreporting of information on drug use, the validity of the questions in the conscript surveys have been assessed previously and found to be satisfactory [32,33]. Further, we investigated the number of hospital admissions with a diagnosis of drug abuse during the follow-up period, and found a high correlation between level of cannabis use at conscription and hospital admission for drug abuse.

## Conclusions

We did not find evidence for an increased risk of depression among subjects with history of cannabis use by age 18 to 20. Our finding that there is an increased risk of schizoaffective disorder related to cannabis use is consistent with previous studies of cannabis and psychosis.

Our results indicate that the association between cannabis use and subsequent severe depression is likely to be confounded by common risk factors for both, such as disturbed behavior during childhood. More research is needed to explore if there is any association between cannabis use and milder forms of depression. However, given the lack of association with severe depression whereas a strong relationship with schizoaffective disorder was found suggests that cannabis use may mainly be associated with psychotic disorders.

## Competing interests

The authors declare that they have no competing interests.

## Authors’ contributions

All the authors contributed to the conception and design of the study, analysis and interpretation of the data, and drafting the paper and revising it critically for key intellectual content. Finally, they all approved the final version.

## Pre-publication history

The pre-publication history for this paper can be accessed here:

http://www.biomedcentral.com/1471-244X/12/112/prepub
